# A Rare Case of Renal Vein Thrombosis Secondary to Oral Contraceptive Pills

**DOI:** 10.7759/cureus.57604

**Published:** 2024-04-04

**Authors:** Lavneet Chawla, Amitoj S Sachdeva

**Affiliations:** 1 Adult Hospitalist Services, OSF Saint Francis Medical Center, Peoria, USA; 2 Internal Medicine, University of Illinois School of Medicine Peoria, Peoria, USA

**Keywords:** venous thromboembolism (vte), direct oral anticoagulant, flank pain, oral contraceptive pill (ocp), renal vein thrombosis

## Abstract

Renal vein thrombosis (RVT) is a common complication of nephrotic syndrome and renal malignancy. However, its association with oral contraceptive use has rarely been reported. We report a case of a 29-year-old female with a history of oral contraceptive use, presenting with acute flank pain. On further investigation, she was found to have unilateral RVT. Oral contraception was discontinued, and she was started on therapeutic anticoagulation, initially with low-molecular-weight heparin, and then switched to apixaban. Her symptoms improved, and she is currently doing well. This case signifies the importance of proper history-taking and how oral contraception should be considered a significant risk factor for venous thromboembolism.

## Introduction

Renal vein thrombosis (RVT) is a relatively rare condition characterized by thrombus formation in the renal veins or their branches. It is commonly seen in patients with nephrotic syndrome, renal malignancy, or in infants with inherited thrombophilia [1]. Previous studies have reported an increased incidence of venous thromboembolism with oral contraceptive pills (OCPs), but solitary RVT has rarely been reported. Here, we present a case of unilateral RVT in a patient taking OCPs for polycystic ovarian syndrome (PCOS) and an unremarkable inherited thrombophilia workup.

## Case presentation

**Figure 1:**
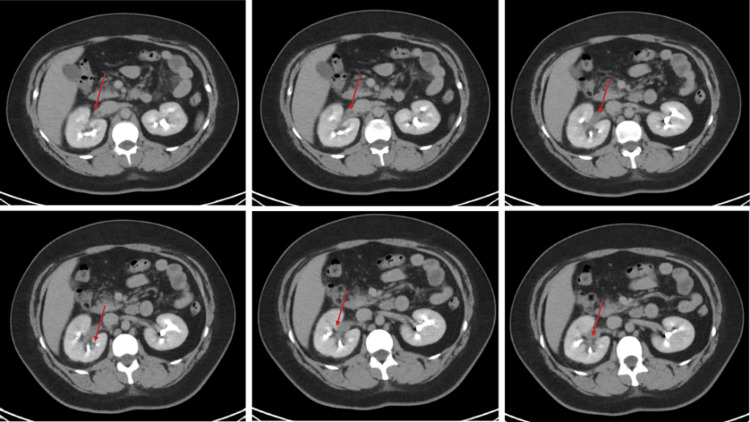
CT of the abdomen and pelvis with an IV contrast series demonstrating mild right perinephric fat stranding and filling defect in the right renal vein (red arrow).

**Figure 2:**
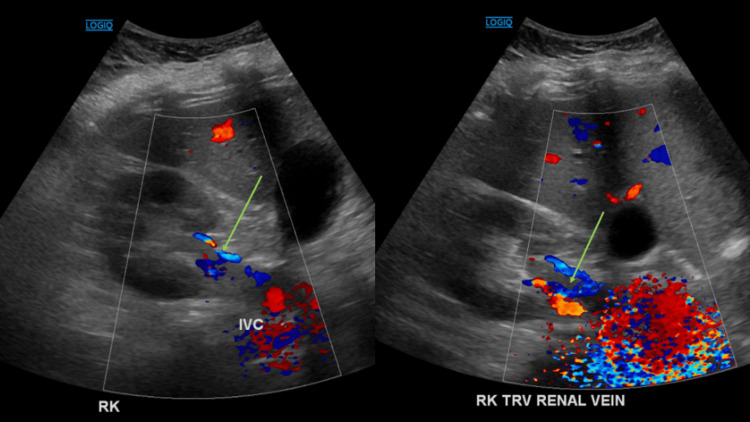
Hyperechogenicity within the right renal vein with elevated venous flow (green arrow) on renal Doppler ultrasound. RK: right kidney; IVC: inferior vena cava; TRV: traverse

A 29-year-old Caucasian female with a previous medical history of iron deficiency anemia (IDA) and long-term estrogen-based OCP use for PCOS presented with sharp right flank pain for two days, radiating down to her right groin. She had associated non-bloody, non-bilious vomiting and suprapubic discomfort. She denied fevers, chills, dysuria, gross hematuria, heavy weightlifting, or trauma to her back. She denied any history of smoking. Her vitals and physical examination were unremarkable. The patient's BMI was 42 kg/m^2^. The complete blood count showed a white blood cell count of 15,350/mcL and a hemoglobin level of 7.8 g/dL. The complete metabolic panel was unremarkable. The urinalysis showed 3+ protein, 11-20 WBC/hpf (high power field), and a moderate amount of bacteria. The D-dimer level was 2.87 mcg/mL (normal <0.50 mcg/mL), and SARS-CoV-2 was negative. A computed tomography (CT) scan of the abdomen and pelvis with contrast showed no renal or ureteral stones. However, it showed mild right perinephric stranding and a filling defect in the right renal vein, concerning a thrombus (Figure 1). The renal Doppler ultrasound showed an area of hyperechogenicity within the right renal vein with elevated venous flow, consistent with thrombosis (Figure 2). OCP was discontinued, and she was started on therapeutic (1 mg/kg every 12 hours) low-molecular-weight heparin (LMWH) for RVT and ceftriaxone for concurrent pyelonephritis. The patient underwent a thrombophilia workup that was unremarkable, including antithrombin III, homocysteine levels, factor V Leiden, factor II prothrombin gene, lupus anticoagulant, and proteins C and S. The patient’s renal function remained stable, and her symptoms improved. She was transitioned to apixaban and referred to gynecology for PCOS management with the recommendation of avoiding estrogen-based therapy and completing a 24-hour urine protein study. 

## Discussion

Apart from OCPs, other risk factors for RVT include infection, trauma, extrarenal compression of the renal vein, and, most recently, SARS-CoV-2 [2]. OCPs affect blood clotting by increasing plasma fibrinogen levels and increasing the activity of factors VII and X. The antithrombin activity is decreased. RVT primarily affects the left side and is more common in women than men. The symptoms of acute RVT may include flank pain, back pain, or hematuria, and if bilateral, patients can have renal dysfunction. Diagnosing RVT requires clinical suspicion and can be confirmed with CT angiography or magnetic resonance (MR) venography. Ultrasonography with Doppler has low sensitivity [3].

In our case, the patient presented with acute flank pain and had a right RVT with no evidence of nephrotic syndrome or malignancy on abdominal imaging, and her thrombophilia workup was unremarkable. OCP use appeared to be her unique underlying risk factor, although underlying pyelonephritis as a risk factor could not be completely ruled out. There have been a few cases of RVT reported previously with OCP use [4-11]. Miyahara et al. reported a case of left RVT in a patient using OCPs. Their patient also had smoking as an additional contributing risk factor, which was not seen in our patient [4]. Sasaki et al. reported a case of left RVT in a Japanese female patient, who also had smoking as an additional contributing risk factor [5].

Local thrombolytic therapy with or without catheter-directed thrombolysis is recommended for patients with renal dysfunction to restore renal venous flow and renal function. In contrast, therapeutic anticoagulation is recommended for patients with chronic RVT or acute RVT without renal dysfunction. The efficacy of warfarin as a direct oral anticoagulant (DOAC) has not been well studied. However, patients should receive unfractionated heparin or LMWH prior to transitioning to warfarin. A few studies have reported the role of DOACs in RVT. Zhang et al. performed a small study on 16 patients and observed similar efficacy with rivaroxaban compared to LMWH [12]. Matta et al. successfully treated a 44-year-old patient with rivaroxaban who presented with isolated right RVT and bilateral pulmonary embolism [13]. In our case, OCP discontinuation and underlying menorrhagia added further complexity as anticoagulation increased the patient's bleeding risk. After a multidisciplinary discussion with the hematologist and the patient, apixaban was used with plans of repeating imaging in three months to monitor thrombus status.

## Conclusions

We highlight the importance of having RVT on the differential diagnosis when a patient presents with acute flank pain, especially with a history of OCP use. This case also demonstrates a therapeutic dilemma due to the limited evidence to support the use of DOACs in RVT, and more studies are needed to establish their use in the treatment of RVT.
